# The Challenges of the Camel Welfare in Western Countries

**DOI:** 10.3390/ani16182929

**Published:** 2026-09-17

**Authors:** Bernard Faye, Laura Menchetti

**Affiliations:** 1 UMR Systèmes d’Elevage en Milieux Méditerranéens et Tropicaux (SELMET), CIRAD-Department Environment and Society (ES), Campus International de Baillarguet, 34398 Montpellier, France; 2 School of Biosciences and Veterinary Medicine, University of Camerino, 62024 Matelica, Italy

**Keywords:** animal welfare, acclimatization, large camelids, farming system, Europe

## Abstract

Camels are increasingly kept in Europe and other Western countries for tourism, leisure, sport, and production, raising questions about their acclimatization to environmental and husbandry conditions differing from traditional camel-rearing regions. This narrative review examines camel presence in Western countries, adaptations to desert environments, and conditions encountered in these new contexts. Potential welfare challenges are then discussed according to the four Welfare Quality^®^ principles, while management and human–animal interactions are considered as cross-cutting factors in accordance with the Five Domains Model. Camels possess remarkable physiological and behavioral adaptations to arid environments. Although some environmental stressors may be attenuated in Western contexts, these conditions do not necessarily translate into better welfare. Richer diets, altered grazing and browsing opportunities, reduced mobility, confinement, social restrictions, novel human–animal interactions, and different climatic and disease pressures may introduce new challenges affecting health, metabolism, and behavior. Ensuring camel welfare in Western environments requires a two-way process: the capacity of camels to acclimatize to these conditions and the ability of owners, caretakers, and veterinarians to recognize and meet their species-specific needs. Further research is needed to optimize husbandry and management practices in these new contexts.

## Simple Summary

Camels are increasingly kept in Europe and other Western countries for tourism, leisure, sport, and, more recently, milk production. This expansion raises questions about whether animals adapted to the climatic, environmental, and husbandry conditions of arid regions can maintain good welfare under different conditions. This review examines current knowledge on camel adaptation and the main welfare challenges associated with their presence in Western countries. Camels possess remarkable physiological and behavioral adaptations to desert environments. In Western countries, some of the environmental challenges they traditionally face, such as extreme heat, water scarcity, and limited feed availability, may be reduced. However, the absence or attenuation of these stressors does not necessarily guarantee good welfare. Differences in pasture and feeding conditions, reduced opportunities for movement and social interactions, inappropriate housing, unfamiliar health threats, and limited camel-specific knowledge among owners and veterinarians may create new welfare concerns. Particular attention should also be given to appropriate handling and training and to the quality of human–camel interactions. Overall, camels appear capable of adapting to Western environments, but ensuring their welfare requires management practices that recognize their species-specific physiological and behavioral needs rather than simply transferring practices developed for other livestock species.

## 1. Introduction

Large camelids are domestic animals considered to be suited to particular desert environments: hot deserts in the Sahara and the Middle East, as far as the western part of India for the dromedary; and cold deserts of Central Asia, from the Caspian Sea to Inner Mongolia in China, for the Bactrian camel. At the biological level, their anatomical and physiological adaptations to the conditions of these deserts are proverbial. Their ability to withstand extreme heat or cold and to cope with limited water availability is a hallmark of their adaptation to these environments. Camels can space out their water intake and subsequently drink large quantities after dehydration, while also using desert fodder known for its low nutritional value. Together with their distinctive anatomical features, these characteristics make them particularly well adapted to the constraints of their environment [1,2,3,4,5,6].

However, in recent decades, camel farming has undergone substantial changes driven by urbanization in traditional camel-rearing regions, demographic pressure, increasing scarcity of pastoral resources, and, more broadly, climate change [7,8,9,10]. These widely documented transformations can be summarized in six main trends: (i) partial or complete, temporary or permanent sedentarization of camel herders [9]; (ii) peri-urbanization of entire herds or of their productive components [10]; (iii) commodification of camel milk, involving a transition from a “gift economy” towards market-oriented or even capitalist production systems [11,12,13]; (iv) intensification of production systems, particularly with regard to feeding and reproduction [14,15]; (v) specialization of production activities, with the emergence of dairy farms, feedlots, and racing stables [14,16,17]; and (vi) diversification of camel-related activities towards tourism and trade [17], together with the increasing involvement in camel farming of people from outside traditional pastoral communities, including traders, civil servants, and political decision-makers [17].

But another important change occurred in recent decades, a change with complex origins: the expansion of camel farming outside its usual ecosystem (arid and semi-arid) and especially in Western countries [17]. Such an expansion raises concerns regarding the welfare of camelids that are naturally adapted to arid environments, including the hot deserts inhabited by dromedaries and the cold deserts inhabited by Bactrian camels. In addition, questions have been raised as to whether existing legislative frameworks adequately address the species-specific welfare requirements of camels [13].

Thus, this review aimed to critically examine the main welfare challenges associated with keeping large camelids in Western countries, with particular emphasis on their adaptation to environments differing from those in which they are traditionally raised and on the implications of feeding, housing, health, behavior, and management practices.

## 2. Materials and Methods

A narrative literature review was conducted between April and August 2026. Relevant literature was progressively identified through searches of PubMed and ScienceDirect, complemented by the authors’ personal collections of scientific books and other relevant literature on camel biology, husbandry, and welfare. Searches were performed iteratively throughout the preparation of the manuscript using combinations of keywords related to the topics addressed, including “camel”, “dromedary”, “Bactrian camel”, “animal welfare”, “adaptation”, “acclimatization”, “housing”, “feeding”, “nutrition”, “health”, “behavior”, and “management”. Given the narrative nature of the review, the identification and selection of relevant literature were also informed by the authors’ longstanding research and professional experience in camel science and welfare, including participation in international scientific meetings and collaborative research activities. Publications were selected according to their relevance to the scope of the review. Eligibility was primarily based on the relevance of the publication to camel biology, adaptation, husbandry, health, behavior, or welfare, with particular attention to sources providing information applicable to the environmental and management conditions addressed in this review. Publications available in English were primarily considered. Original research articles and review papers were considered, together with scientific books, book chapters, conference proceedings, and relevant institutional or technical publications when they provided pertinent camel-specific information. Publications were considered on the basis of the complete available source rather than on bibliographic records alone. When the same publication was identified through multiple searches or sources, it was considered only once. No restrictions were applied regarding publication year, given the need to include both historical and contemporary literature relevant to camel biology and husbandry.

The review was structured in two main parts. The first provides the contextual basis for the welfare discussion by examining the historical and current presence of camels in Western countries, their adaptation mechanisms to desert environments, and the environmental and husbandry conditions encountered in these new contexts. The second focuses on the potential welfare challenges associated with these conditions. To structure this discussion, reference was made to the four welfare principles established within the Welfare Quality^®^ framework: Good Feeding, Good Housing, Good Health, and Appropriate Behavior [18]. These principles have been applied and adapted in several key publications addressing camel welfare assessment and management across different husbandry systems [19,20,21,22,23] and were therefore considered appropriate for structuring the present review. The Five Domains Model [24] was also considered as a complementary conceptual framework, particularly to account for behavioral interactions with the environment, conspecifics, and humans, and their potential contribution to both negative and positive welfare experiences. For each welfare challenge identified, practical recommendations are provided to support its prevention or mitigation under Western husbandry conditions.

## 3. Camels in Western Countries: A Novelty?

Although the use of large camelids in recent decades, particularly in Europe [25], for the development of livestock farming for tourism, sports, or agricultural purposes is considered a novelty, their presence on the continent dates back to antiquity [26]. Widely present during the Roman Empire for carrying goods along the Roman ways [27], the use of camels declined in the Middle Ages, except in Spain under Muslim rule, and almost disappeared at the time of the Renaissance. Later, the camel became an object of curiosity in private menageries and royal gardens [28,29].

Indeed, this European presence during the recent historical period almost never had a utilitarian function. Bactrian camels and dromedaries were mainly kept for display, first to the aristocratic elite and, from the nineteenth century onwards, to the general public. Their keeping conditions reflected the animal welfare considerations of the time, and their presence was viewed primarily in terms of acclimatization rather than agricultural use [30]. In brief, until the aftermath of the Second World War, large camelids in Europe were regarded primarily as exotic animals rather than as livestock with a potential role in the diversification of farming activities [31].

In contrast, what can be considered a novelty in Europe in recent decades, particularly since the 1960s, is the changing status of large camelids. From exotic animals mainly confined to zoological parks and circuses, dromedaries and Bactrian camels have increasingly been used for tourism, sport, and production [25]. This transition has involved a shift from settings in which animals were primarily kept for public display to farming and working contexts requiring more complex interactions with owners, handlers, and the public. Consequently, new welfare concerns have emerged, as camels are no longer simply expected to “be” but increasingly to “do”, resulting in new forms of human–animal relationships.

It should be noted, however, that at the European scale, the Canary Islands (Spain) represent an exception, having maintained a camel population used for agricultural purposes for several centuries [32,33].

This point, which is well documented, does not alter the importance of the change in the status of these animals across the rest of Europe. Moreover, even in the Canary Islands, an important change occurred from the 1960s onwards, with the role of camels progressively shifting from agricultural work towards tourism-related activities [34].

Finally, from an animal welfare perspective, the increasing presence of camels in Europe and other Western countries with temperate climates raises two main concerns: (i) the ability of an animal adapted to arid and semi-arid climates to acclimatize to these new environmental conditions; and (ii) the suitability of breeding and management conditions associated with their emerging uses, particularly when camels are kept by people with limited prior knowledge and experience of the species.

## 4. Camels’ Welfare and Acclimatization

Formally, acclimatization refers to the process by which an individual animal gradually adjusts, through physiological and behavioral changes, to new environmental conditions [35]. Thus, the question of acclimatization for a camel has two faces: (i) Can camels adapt to environmental conditions to which the species is not accustomed? (ii) Can camels be introduced into temperate environments without compromising their welfare? Addressing these questions first requires consideration of two aspects: the main biological mechanisms underlying camel adaptation to desert environments and the overall characteristics of their environments of origin and those into which they are introduced.

### 4.1. Desert Environmental Challenges and Camel Adaptations

In camels, each physiological mechanism of adaptation to the desert context, taken in isolation, provides only a small comparative advantage, but it is the set of these mechanisms, coupled with the animal’s anatomical and behavioral particularities, that confers on it an undeniable superiority over other domestic species [36].

The environmental conditions characterizing desert ecosystems can be summarized according to several major features:

(i) Climatic conditions, characterized by marked nychthemeral temperature fluctuations, potentially extreme temperatures—particularly heat for the dromedary and cold for the Bactrian camel—scarce rainfall, low air humidity, dry and hot winds, and intense solar radiation, with UV indices reaching up to 12 [37];
(ii) Scarce and spatially dispersed pastoral resources, mainly consisting of xerophytic plants adapted to arid conditions but generally characterized by low nutritional value, including high lignification and relatively low protein content [38,39,40];
(iii) Limited and spatially dispersed surface water resources, further constrained by high evapotranspiration and resulting in long intervals between opportunities for watering [2,41];
(iv) Limited access to technical services and veterinary care, particularly in highly mobile pastoral systems [42].

Confronted with such conditions, camel adaptation can be summarized as follows. In response to heat stress and marked temperature fluctuations, and unlike small desert animals that can seek refuge underground during the hottest hours of the day, camels have developed a range of thermoregulatory mechanisms aimed at limiting heat gain and promoting heat dissipation. Among these, camels have a well-developed selective brain-cooling mechanism, supported by an extensive carotid rete that functions as a vascular heat exchanger. This system allows arterial blood directed towards the brain to be cooled by venous blood returning from evaporatively cooled surfaces of the nasal cavity, thereby helping to protect the brain from excessive heat [43]. Moreover, the characteristic urination posture of camels, whereby urine may wet the medial surfaces of the hind limbs, has been described as an additional thermoregulatory adaptation, as evaporation of urine from the wetted surfaces may contribute to heat dissipation [6,14]. The concentration of fat reserves in the hump represents another important adaptation to hot environments [44]. By concentrating adipose tissue in a restricted body region rather than distributing it extensively beneath the skin, heat dissipation from the remaining body surface is facilitated. Camels can also reduce metabolic heat production during periods of high environmental temperature. This response, associated with changes in thyroid activity and reductions in heart and respiratory rates [45], decreases oxygen consumption and endogenous heat production, thereby contributing to their ability to tolerate relatively large fluctuations in body temperature in relation to environmental conditions [46].

In addition, other anatomical particularities (long legs and a sternal pad, thick skin, seasonal hair growth) and behavioral characteristics (sitting with the face to the sun, browsing in the shade in the middle of the day, limiting mobility at the hottest time) give camels an undeniable advantage in the desert. A recent study has shown that a camel-specific exon could play a pivotal role in adaptation to extreme environmental stress, particularly in the heart [47].

The adaptation to water scarcity employs comparable metabolic processes through two main mechanisms: water economy by limiting water losses and the maintenance of blood homeostasis in the dehydration/rehydration cycle [3]. From this perspective, several physiological adaptations contribute to the remarkable ability of camels to cope with dehydration and subsequent rehydration, particularly those involving renal function and the distinctive characteristics of red blood cells [48,49]. Renal water conservation is supported by a well-developed renal medulla and a high proportion of nephrons with long loops of Henle, which enhance the kidney’s ability to concentrate urine. In addition, dehydration markedly stimulates arginine vasopressin (AVP) secretion, promoting water reabsorption in the collecting ducts and further reducing urinary water loss [3,48]. At the same time, the distinctive elliptical red blood cells of camels are highly resistant to osmotic stress and can undergo substantial volume changes without hemolysis, contributing to the maintenance of blood flow during dehydration and allowing rapid rehydration without compromising erythrocyte integrity [48,49]. As a result, the water turnover in dromedaries is particularly low (50–100 mL/kg/d vs. 70–230 mL/kg/d for zebu), although the percentage of body water is higher: 72 vs. 60–65% in other domestic herbivore species [50]. Overall, these adaptations allow camels to tolerate substantial changes in body fluid volume and osmolality and to rapidly consume large quantities of water after periods of deprivation without adverse effects [48].

At the cellular and molecular levels, adaptation to environmental stress relies on multiple protective mechanisms, including anti-stress responses, modulation of genes associated with fat metabolism, and heat-shock protein and antioxidant defense systems, which collectively help maintain cellular homeostasis and limit oxidative damage under challenging environmental conditions [3,51].

Camel adaptation is also remarkable in its ability to cope with the low nutritional value and high lignification of many desert plants. This relies on both physiological and behavioral adaptations, including: (i) at the physiological level, a high rate of urea recycling (approximately 94–97%), which enhances microbial protein synthesis in the first compartment [52]; and, at the behavioral level, (ii) ambulatory grazing [53], with animals able to travel more than 20 km per day while foraging; (iii) the consumption of a wider variety of plant species than other ruminants, encompassing the different vegetation strata of their environment, from grasses and shrubs to trees [54]; and (iv) selective feeding behavior, whereby camels preferentially consume certain plant species and consequently spend more time feeding on preferred plants [55]. Moreover, the characteristics of the salivary glands and oral anatomy facilitate the chewing and ingestion of thorny and highly lignified plants [56]. Together, these adaptations enable camels to efficiently exploit the scarce and low-quality nutritional resources available in desert environments [49].

Finally, although camels are susceptible to a wide range of infectious diseases, they have been reported to show comparatively high resistance to some pathogens affecting other livestock species. Resistance to diseases such as tetanus and bovine spongiform encephalopathy has been reported, although the available evidence remains limited [57]. A more consistently documented example is foot-and-mouth disease, to which dromedary camels appear to have very low susceptibility under experimental conditions, unlike cattle and Bactrian camels [58]. Similar observations were reported regarding the lack of sensitivity or low sensitivity of camels to the main infectious diseases in tropical countries, such as rinderpest or Bovine Contagious Peri-Pneumonia (PCPP). Camelids also possess distinctive features of the adaptive immune system, including unconventional heavy-chain-only antibodies. Recent evidence indicates that this distinctive antibody architecture also extends to the IgA isotype in dromedaries, potentially contributing to mucosal immune defense [59].

Thus, the entire physiology, metabolism, and behavior of the camel appear to be focused on resistance to the constraints of the desert environment. These particularities may represent either an advantage or a potential hindrance when camels are introduced into environments outside their native range, depending on the environmental and management conditions encountered.

### 4.2. Living Conditions of Camels in Their New Environments

To understand the process of acclimatization, it is also necessary to consider the environmental and management conditions that camels encounter in their new context. Climatic conditions vary considerably across Europe, from the Mediterranean climate of southern regions to the oceanic climate of western Europe and the more continental climate of central Europe. Nevertheless, compared with the environments of origin of most camel populations, temperatures are generally more moderate, while humidity levels may be considerably higher. Another important factor is the higher nutritional value and greater availability of natural forage in temperate regions compared with tropical and, particularly, desert pastures [60]. The sanitary context also differs, with a dense veterinary network and generally easier access to veterinary services, medicines, and preventive care.

At first sight, these climatic, nutritional, and sanitary conditions may therefore appear more favorable to animal welfare than those prevailing in the environments of origin. However, apparently favorable environmental conditions do not necessarily translate into improved welfare. When camels are introduced into these new contexts, they may encounter novel physiological, behavioral, and management challenges that require specific adaptations, despite the potentially greater environmental comfort.

## 5. Camel Welfare and Adaptation to the New Environment

Animal welfare is multidimensional and cannot be adequately characterized by a single measure. In a pioneering camel welfare study, Menchetti et al. [61] emphasized the need for reliable animal-based measures to assess welfare across its different dimensions. Accordingly, the following sections discuss the main welfare challenges associated with camel keeping in Western countries according to the four welfare principles of the Welfare Quality^®^ framework [18]—Good Feeding, Good Housing, Good Health, and Appropriate Behavior—while particular attention is also given to management and human–animal interactions as cross-cutting welfare factors, consistent with the Five Domains Model and its emphasis on animals’ interactions with their environment, other animals, and humans [24].

### 5.1. Good Feeding for Camels in Western Countries

The question of good camel feeding in the European context concerns two aspects: outdoor pasture feeding and indoor supplementary feeding. Camels are herbivores and, as such, use pastoral areas. However, pastures in temperate latitudes differ considerably from rangelands in desert areas, both in terms of floristic composition and nutritive value. As described above, camels have developed a range of physiological and behavioral adaptations that allow them to efficiently exploit the scarce and spatially dispersed nutritional resources of arid environments [50,53,54,55].

**Figure 1:**
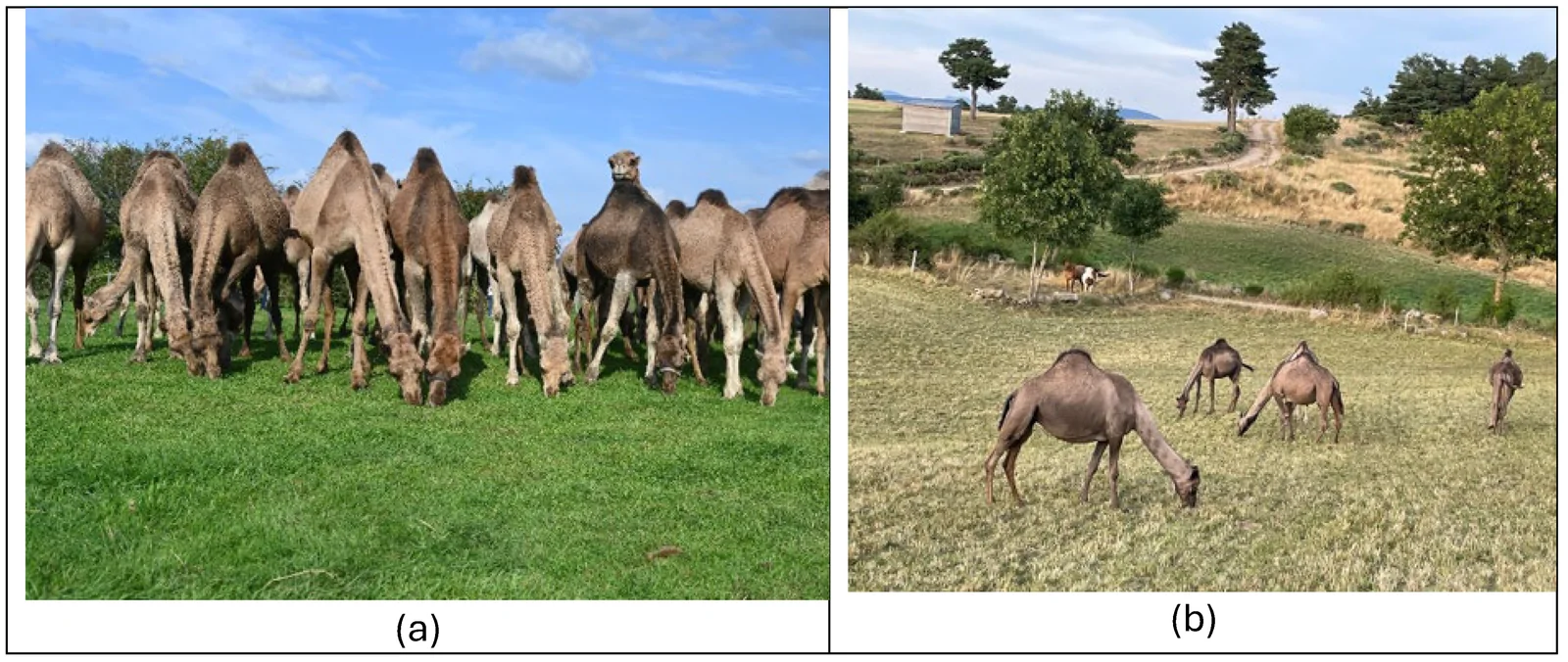
Camels grazing in France: (**a**) in northern France during spring; (**b**) in central France during summer (© B. Faye).

In Europe, especially in areas with more oceanic or continental climates, pastures, even natural ones, are characterized by higher nutritional value and much higher productivity per hectare. Such a grazing context is likely to disrupt the camel’s innate behavior on pasture, as it can meet its nutritional requirements in a very short time. While camels may spend more than 8 h per day walking and foraging across Saharan rangelands to meet their nutritional needs, the same requirements can be fulfilled in temperate pastures in considerably less time, with less movement and lower energy expenditure (Figure 1). However, what may initially appear to represent more favorable feeding conditions may also pose risks to animal welfare, particularly due to access to excessively rich diets, especially spring grass [22,62,63,64], and to the reduced time spent expressing motivated locomotor and foraging behaviors. Whereas in their environment of origin the main nutritional risks for camels are dietary deficiencies or imbalances in protein, energy, and minerals, in temperate environments, characterized by abundant, nutrient-rich pastures and often lower floristic diversity, the risks of metabolic disorders and obesity may increase, as suggested by observations from some camel farms in France [64].

Such a risk is further accentuated in indoor systems. Firstly, indoor housing physically restricts or prevents access to grazing and browsing. Indeed, intensively housed camels have been reported to spend less time feeding and very little time walking [65], potentially limiting opportunities to express highly motivated foraging and locomotor behaviors, with consequences for their welfare [66] (see Section 5.4). Differences in plasma cortisol concentrations have been reported between farming systems, with higher levels in farm-kept camels than in those maintained under pastoral grazing conditions; lower post-parturition cortisol concentrations were also observed in the pastoral system [67]. Secondly, to compensate for the restricted access to natural vegetation, feed supplements are commonly provided to the camels. However, the complementary feeds distributed to the animals have high energy and protein concentrations, which further increase metabolic risks and can lead to serious health consequences [63,64].

Regarding watering, we can assume effortlessly that access to water in Europe is easier than in desert areas. However, the ability of the camel to go without drinking for long periods of time does not imply that it should be deprived of water. Although its water requirements are lower than other species, regular watering should be ensured, especially during the summertime [20,68].

**Figure 2:**
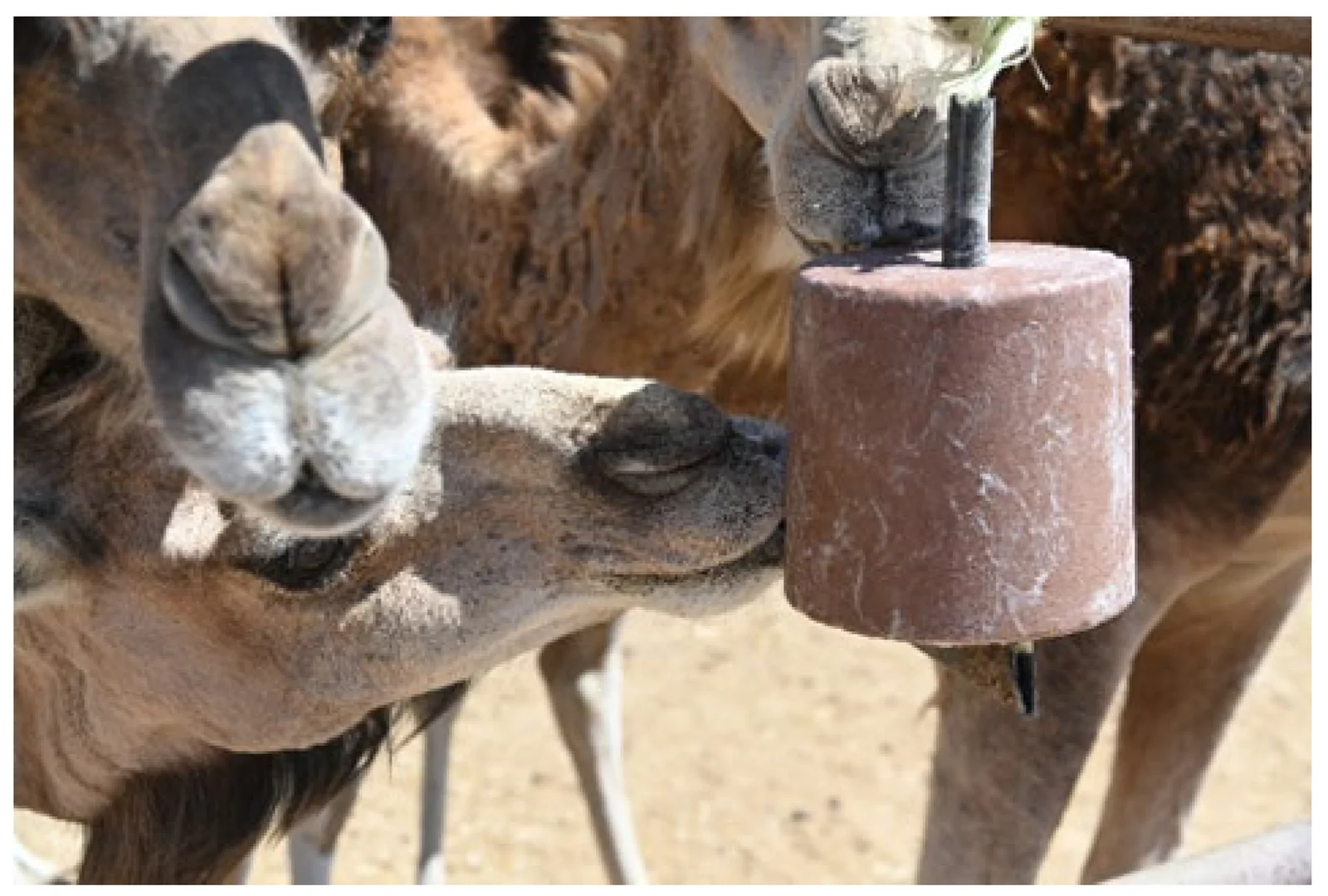
Providing mineral complement to the camel (© B. Faye).

**Figure 3:**
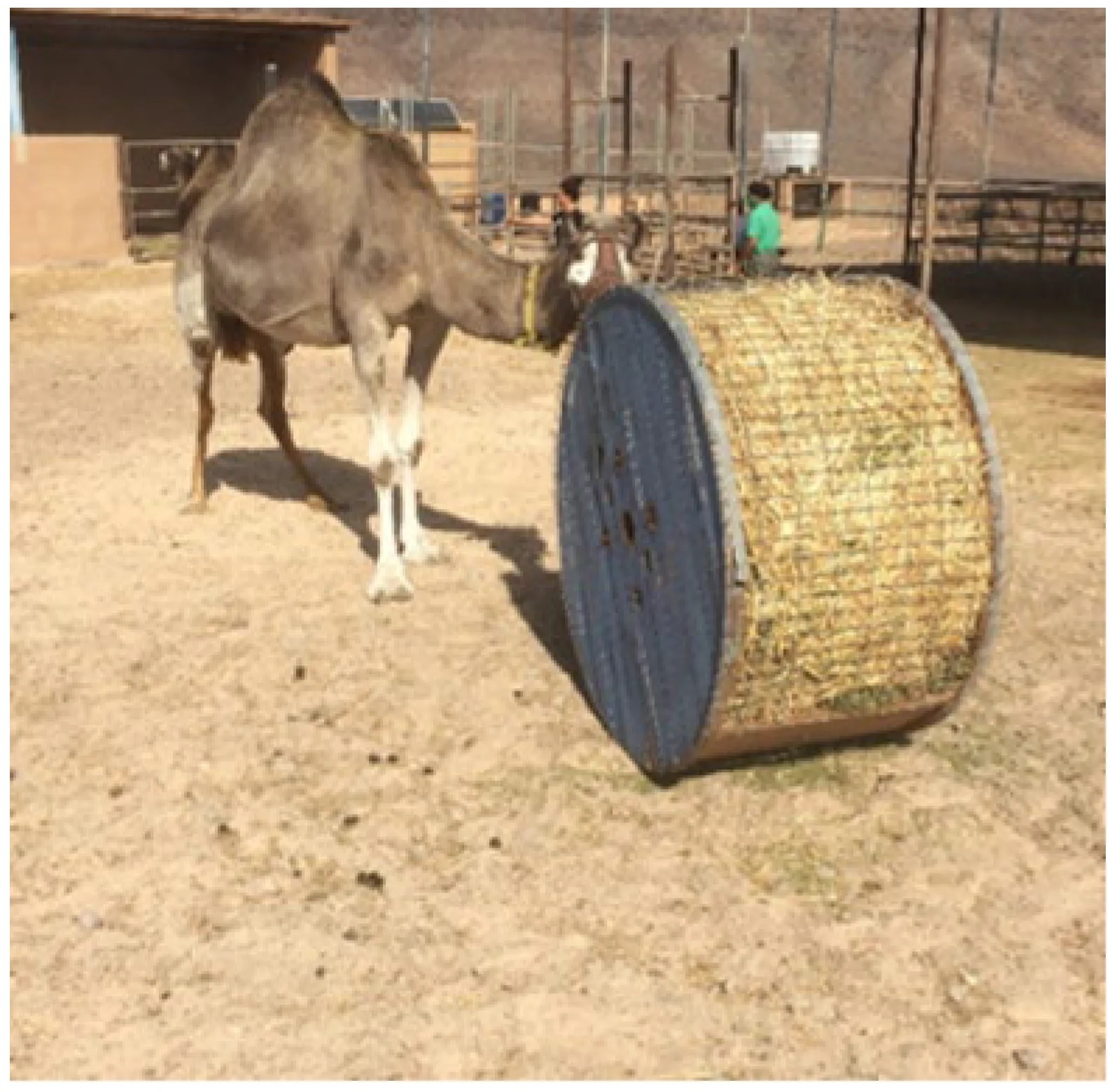
Distribution of hay in a metallic net to increase the feeding time (© B. Faye).

Thus, the main welfare criteria to be retained for the camel feeding would be:

1. Give preference to natural rangelands, even if they are of poor quality (pastures overgrown with thistles, nettles or brambles are a delight for camels).
2. Limit grazing time, especially in early spring when the grass is too rich, and bring hay and straw to the pens before leaving for grazing [63,64]; alternatively, the limited grazing can be obtained by a high animal density in pasture.
3. Be careful in the use of feed supplements that are too concentrated in energy and protein, intended for other species such as young bulls for fattening, or dairy cows; preferably give supplements intended for horses bred in extensive systems, as specific camel supplements are not available in Europe.
4. Do not forget mineral supplements (Figure 2), especially salt, which camels are fond of; in the desert, camels are major consumers of halophytes [69].
5. Monitor the body condition of the animals, the optimal score being between 3 and 4 on a scale of 0 to 5, both in the dromedary and in the Bactrian [20,70], especially at key moments in the physiological cycle (end of gestation, beginning of lactation, mating).
6. In case of an indoor system, at least in the winter season, it is possible to envisage systems that force the animals to spend more time on the feeding function, such as enclosing the basal diet (hay) in nets, making access less easy (Figure 3).
7. Provide access to clean water regularly, despite the reputation of the camel for resistance to water deprivation.

### 5.2. Good Housing for Camels in Western Countries

In pastoral systems from drylands, camels generally do not have access to livestock housing. In most cases, the animals are brought home at night in pens made of thorny plants, or sometimes in pens made of metal materials. During transhumance, it is even common to have no shelter of any kind, the animals being simply hobbled to prevent them from dispersing too far during the night. Solid buildings, generally open to all winds, and with or without a roof to provide shade for the hottest hours, are proposed in more intensive systems.

The main issue in temperate environments is not so much protection against heat as against humidity. This therefore implies the possibility of building closed structures so that the camels can protect themselves from bad weather, especially rain.

Unlike several other farm animal species, for which European legislation establishes specific minimum space allowances under particular production systems, no harmonized species-specific minimum indoor space requirements have been established for camels at the European level. However, it is generally accepted that each camel should have a minimum indoor space allowance of 15–20 m^2^, with a roof height approximately twice the height of the animal at the top of the head [62]. Unlike in desert regions, where camel shelters commonly have sand or earthen floors, housing in temperate regions may have concrete or earthen floors covered with straw bedding. No harmonized European standards currently define specific bedding requirements for camels. Because camel feces are generally drier and less abundant than those of cattle, bedding requirements may be lower. In their book on camel farming management, Faye et al. [62] suggested, as a practical recommendation, the use of approximately 3–5 kg of straw per camel per day, based on quantities previously recommended for dairy cattle [71]. Nevertheless, under the wetter climatic conditions typical of many Western countries, particular attention should be paid to flooring and drainage, ensuring that housing and outdoor areas provide dry, well-drained, non-slip surfaces and minimize prolonged exposure to wet or muddy ground [22]. 

**Figure 4:**
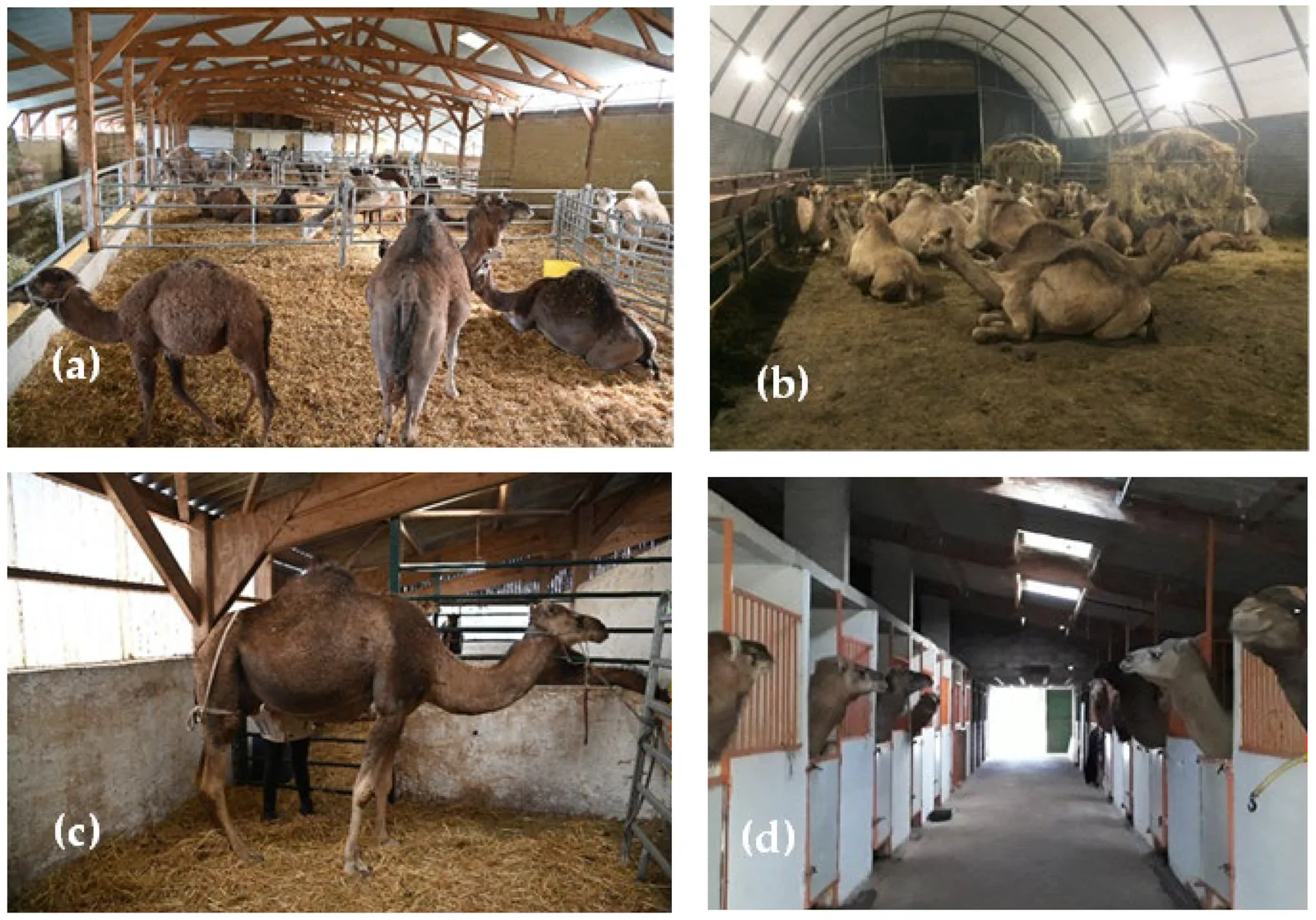
Some types of camel housing in Europe: (**a**) modern cowsheds with paddocks; (**b**) tunnel structure; (**c**) old agricultural building; (**d**) boxes for horses (© B. Faye and Dromasud).

A wide variety of camel housing types can be seen in Europe, from modern cowsheds comparable to those of cattle (Netherlands, Germany, Center of France), tunnel structures (northern France), boxes identical to those of horses (southern France), or simple repurposing of old agricultural buildings (Figure 4). However, housing design should not only provide protection from adverse weather conditions but also allow camels to express important behavioral needs, particularly locomotor and social activities. Under traditional pastoral systems, camels are generally managed in herds, providing ample opportunities for locomotion, grazing, and social interaction. Herd composition may include females at different physiological stages, whereas breeding males may be managed separately and introduced into the herd during the reproductive season [21,72]. In modern intensive camel farming systems, group housing tends to be more structured. Although there is no universal classification of groups applicable across all farms, animals may be grouped according to age, physiological status, production level, and genetic potential. This organization also facilitates the implementation of feeding programs tailored to the specific nutritional requirements of each category [14]. Breeding males are generally housed individually, particularly during the rutting season, because of the increased risk of aggressive interactions. However, prolonged confinement in individual boxes has been associated with the expression of stereotypic behaviors in male dromedaries, whereas providing opportunities for free movement and social contact reduced their occurrence [73] (see Section 5.4). Particular attention should also be paid to calf management because of the strong bond between the dam and her offspring. Physical and visual contact between the dam and calf is important for maternal behavior and milk let-down [74,75,76]. In intensive dairy systems, calves may therefore be maintained in adjacent pens, allowing contact with their dams while controlling suckling and supporting milk production. In addition, because all young camels are born in the same season due to the seasonality of the reproductive cycle, their gathering in the same paddock forms cohorts that facilitate socialization and foster inter-individual interactions [14].

In the absence of a structured building, totally or partially closed, as is the case in Spain, for example, it is important to have an area sheltered from the sun. Despite their well-known adaptation to high environmental temperatures, access to shade appears to be relevant for camel welfare. Camels may still exhibit physiological responses to seasonal heat, with higher blood cortisol concentrations reported during summer than in winter [77]. Moreover, camels provided with adequate shaded areas spend more time in recumbency and rumination than those without access to shade, suggesting that shade may facilitate resting and comfort-related behaviors [78].

Based on the available literature and the considerations discussed above, the main housing recommendations for maintaining camel welfare can be summarized as follows:

1. A minimum floor area of approximately 15 m^2^ per adult camel;
2. A roof height of approximately 5 m;
3. Protection from prevailing winds through appropriately positioned solid walls;
4. A fully covered resting area providing adequate protection from direct solar radiation and sufficient shade [78];
5. Adequate ventilation to maintain good air quality, although camelids have been reported to produce approximately 44% less methane than ruminants when expressed per unit of body mass and approximately 27% less when expressed per unit of dry matter intake [79] and may generate lower ammonia emissions [80];
6. Solid barriers and partitions without protruding or potentially injurious elements;
7. Dry bedding and well-drained flooring [19,22];
8. An appropriate light–dark cycle, avoiding artificial lighting during the night;
9. A separate area for sick animals and newly introduced animals requiring isolation or quarantine;
10. Free access to drinking water and appropriate feed;
11. Feeding areas designed to protect forage from rain and excessive moisture;
12. Access to a sufficiently large outdoor exercise area allowing free movement.

### 5.3. Good Health for Camels: New Challenges?

Good health is an essential part of welfare. From this point of view, the challenges in camel farms established in Europe can be summarized in two points: (i) the particularity of the health patterns compared to the dominant diseases in arid zones, and (ii) the limited camel-specific experience and training currently available to many veterinary practitioners in Western countries.

The main health disorders in camels have long been known and documented [81], the first camel scientists being veterinarians. Trypanosomosis (surra), mange, calf diarrhea, and gastrointestinal parasites are among the most common diseases, sometimes affecting entire herds [82]. In addition, a comprehensive list of infectious and parasitic diseases affecting dromedaries and Bactrians was established by the expert ad hoc group on “camelid diseases” at the World Organization of Animal Health (WOAH) and subsequently published in an official book [83]. For both dromedary and Bactrian camel, 11 viral diseases, 13 bacterial diseases, and 9 parasitic diseases were described. Thus, camelpox, brucellosis, contagious ecthyma, mastitis, respiratory diseases, and tick-borne diseases, among others, continue to represent major constraints to productivity and welfare in camel-producing countries, notably in pastoral areas [84,85].

Some of these diseases can be introduced into European camel herds. For example, in camels imported from the Canary Islands (Spain), trypanosomosis due to *T. evansi* was introduced for the first time in France, resulting in the death of one animal and a prolonged course of treatment with Melarsomine [86]. However, with access to veterinary medicine being easier in Europe, the risks linked to such introduction are globally limited. Moreover, the import of camels from outside the Schengen area is usually not permitted for sanitary reasons. If such a decision increases the risk of consanguinity in the European herd, it limits the risk of introducing infectious diseases. For example, the risk of MERS emergence in Europe appears more linked to potentially contaminated humans arriving from the Middle East than to camels [87].

In the context of intensifying production systems, especially in the Gulf countries, camels are facing new health challenges, whether in intensive dairy systems, in feedlots for fattening, or in racing stables. Diseases such as mastitis, infertility, acidosis, or mineral imbalances have become more dominant [14,88,89]. While these health disorders, and the challenges concerning the associated well-being, can be related to the new conditions of camel breeding in these intensive systems, climate change and geographical expansion of camels under extensive systems, particularly in sub-Saharan Africa, are implicated in the emergence of new infectious diseases with often ill-defined etiologies [90,91,92]. It is also in this context of multidimensional changes that the emergence of MERS-CoV as the main zoonosis transmitted by camels since 2012 seems to be taking place [93].

However, the European context seems different. The intensification of camel production is driven primarily by greater access to abundant, nutrient-rich feed resources rather than by efforts to enhance dairy production, sports performance, or growth. Unfortunately, there are few studies on the dominant diseases on camel farms in Western countries. In one of the few health monitoring studies carried out in France involving biological analyses and autopsies [63,64], it emerged that 3 major causes of mortality could be retained: specific mineral deficits, in particular selenium, uremic toxicity due to excessive dietary nitrogen, and climatic stress in young animals born in cold/wet times. Some of the most common symptoms included emaciation, acidosis, and diarrhea. In addition, blood biochemical analyses (in particular liver and kidney enzymes) suggested that the main disorders were of metabolic origin, leading to liver and kidney failure. In addition, more recent observations suggest that we are witnessing effects linked to inbreeding in the European population, mainly stemming from a small population originating in the Canary Islands, with consequences such as congenital fragility, limb conformation abnormalities, or hoof deviation due to wear [62].

But beyond health problems, such farming conditions, which result in reduced mobility, changes in diets, winter confinement in closed buildings, and more frequent camel handling, can lead to behavioral problems [94] (see Section 5.4), which should be regarded as a component of preventive veterinary medicine, rather than an independent welfare issue only [95]. Thus, to achieve “good health”, it is not enough to ensure appropriate prevention (vaccination, systematic deworming). It is also necessary to ensure “good management” that promotes positive human–camel interactions and an environment that facilitates them. Indeed, management practices and caretakers’ experience have been associated with the occurrence and recognition of health problems in camels, highlighting the importance of camel-specific knowledge and appropriate veterinary support [96] (see Section 5.5). One of the major issues from this point of view is the competence of veterinarians in terms of camel disease know-how.

Few practitioners in Europe are confronted with these animals among their clientele and, in most cases, are unaware of their physiology and diseases, know nothing about restraint techniques or blood standards, or use veterinary medicine that is not necessarily adapted to camels, to take just a few examples. Training sessions were organized through private initiatives (for example, by the European Camel Ranch Owners Association (ECROA) in Europe, or the North American Camel Ranch Owners Association (NACROA) in the USA), but this is still insufficient, especially since no institutional support is currently available. For example, in France, no training has been provided by the official Veterinary Technical Groups (GTV) and camel-specific training appears to remain limited in the curricula of many European veterinary schools. There are many scientific and extension books on camel diseases, but these are mainly intended for camel owners and vets in the “camel countries” (e.g., [81]) and not for an audience of European and American breeders. The recent book by Faye et al. [62], translated into French, Arabic, Spanish, Turkish and Russian, aimed to partially compensate for this lack.

Overall, there is a need to adopt a more integrative approach to camel health, given its multifactorial nature. The notion of “Good Health” is firmly intertwined with that of the right environment, adequate nutrition, the fulfillment of species-specific behavioral needs, and informed management to ensure the overall well-being of camels in their new context. It is therefore also the question of behavior and management that must be addressed now.

### 5.4. Appropriate Behavior for Camels in Western Countries

Camels have a complex behavioral repertoire including feeding and foraging, rumination, resting, locomotion, social interactions, and reproductive behaviors [22,97]. Under natural and pastoral conditions, camels spend a considerable part of the day moving and foraging over large areas and normally live in socially structured groups, with a particularly strong bond between dams and their offspring. The opportunity to express these species-specific behaviors is therefore an important component of their welfare.

From a behavioral perspective, the main welfare challenges for camels kept in Western countries are likely to arise from two main aspects: changes in husbandry and feeding conditions compared with traditional pastoral systems, and the quality of human–animal interactions, the latter being specifically addressed in the following section. As discussed above, more confined husbandry systems and differences in pasture and diet characteristics may have not only physiological and nutritional consequences but also behavioral implications. These conditions may limit opportunities to express species-specific behaviors, particularly locomotion and social interactions with conspecifics, or alter grazing and foraging patterns. In confined systems, environmental enrichment, including strategies that prolong feeding time (Figure 3) and provide opportunities for browsing, exploration, and rubbing, may therefore help mitigate some of these behavioral limitations [22].

Among the social interactions, the strong dam–calf bond deserves particular attention. Experimental separation–reunion observations have shown marked maternal responses following short-term separation, while other studies have highlighted the importance of calf presence and suckling in stimulating maternal physiological responses, including oxytocin release [74,75,76]. Thus, appropriate management of mother–young interactions should consider their behavioral and physiological consequences, with the dual aim of minimizing potential negative affective states and optimizing production outcomes.

Restrictions on social interactions with conspecifics may represent a broader welfare concern. Evidence from different housing systems indicates that prolonged individual housing with restricted opportunities for movement and social contact can be associated with locomotor stereotypies, such as head-shaking and pacing, and oral stereotypies, including self-biting and bar-mouthing, while providing opportunities for movement and social contact can reduce some of these behavioral alterations [73,98,99]. Studies conducted under different management conditions further indicate that these factors can markedly influence the camel’s daily time budget, affecting the time devoted to walking, feeding, rumination, and resting [65,73,99,100]. Such alterations in the time budget may restrict the expression of highly motivated behaviors, potentially resulting in frustration, contributing to the development of abnormal behaviors, and ultimately compromising animal welfare.

Consistent with these findings, camel welfare assessment protocols developed for both intensive/semi-intensive and pastoral systems consider social interactions and the expression of normal species-specific activities as key indicators of Appropriate Behavior and, more broadly, of camel welfare [20,21].

However, specific knowledge of how camel behavior changes in response to Western husbandry and environmental conditions remains very limited. Available evidence indicates that camels can adapt to novel management procedures, as demonstrated during adaptation to machine milking [101], and can acquire new behaviors through positive-reinforcement-based training, as recently shown for self-loading and self-unloading during transport [102]. These studies, however, concern responses to specific management procedures rather than longer-term adaptation to Western environments. Studies of leisure dromedaries already established in Southern Spain provide some additional insight into camel behavior within Western human-managed environments, revealing substantial individual variation in behavioral traits and coping strategies [103,104]. These differences in coping styles may also reflect individual variation in the capacity to cope with environmental and management challenges, underscoring the importance of monitoring animals individually when assessing their acclimatization and welfare under conditions that differ from those of their origin.

However, these studies did not specifically investigate whether behavioral patterns under Western conditions differ from those observed in camels under comparable management in traditional camel-rearing regions, a key knowledge gap.

Based on the available evidence, ensuring Appropriate Behavior in camels kept in Western countries should therefore include:

1. Providing sufficient opportunities for locomotion and exploration, avoiding prolonged confinement whenever possible;
2. Allowing the expression of feeding and foraging behaviors, including adequate time for feeding and rumination;
3. Providing opportunities for social interactions with conspecifics, while avoiding prolonged social isolation;
4. Ensuring appropriate management of dam–calf relationships to minimize potential negative affective states;
5. Providing appropriate conditions for rest and other species-specific behaviors;
6. Regularly monitoring the behavioral repertoire for the occurrence of stereotypic or other abnormal behaviors, excessive aggression, or marked inactivity, which may indicate that the environment is not adequately meeting the animals’ behavioral needs;
7. Ensuring that owners and caretakers have adequate knowledge of camel ethology and are trained to recognize behavioral changes indicative of pain, discomfort, stress, or disease, particularly because signs of compromised welfare may be subtle and difficult to detect in these animals.

### 5.5. Good Camel Management to Ensure the Well-Being of Animals and Owners

The main factor in the good management of a camel farm is the anthropogenic factor: human expectations, objectives, behavior, and know-how. The camel is a large animal, of significant weight, potentially dangerous if it is not properly handled, especially if it is a male during the period of sexual activity (rut), which is generally in winter [73]. At the same time, well-managed, it is an animal that can show great affection for humans in general and its owner in particular. It is an intelligent animal, capable of acquiring the behaviors expected of it, but is reluctant to change too abruptly.

While attention is often paid to the experience of veterinary practitioners in managing the health of large camelids, equal consideration should be given to the knowledge and skills of camel owners, breeders, and keepers. Indeed, appropriate knowledge of camel behavior and needs, together with adequate management and handling skills, is essential to ensure animal welfare and, particularly, to prevent accidents [96,97,105]. The acquisition of a camel by a private individual does not always involve experienced breeders intending to use the animal for production, tourism, or sporting purposes. In some cases, it may simply result from “love at first sight”, with the camel being regarded primarily as a pet. A camel is not a dog. Understanding camel needs and behavior, recognizing behavioral differences between dromedary and Bactrian camels, and knowing how to handle them appropriately for transport, riding, milking, public presentation, or medical procedures require owners to acquire adequate knowledge and practical skills through appropriate training. This is important not only for human safety and animal manageability, but also for camel welfare, as inappropriate or coercive handling and insufficient habituation to human contact and routine procedures may induce fear and stress, potentially compromising the quality of human–animal interactions [19,97]. Such training is provided in the United States or in Europe within the framework of the associations mentioned above, but remains a private, non-compulsory initiative, and there is no equivalent of an official “certificate of knowledge” at the European level, as exists for horses. There is also no regulatory framework that includes a purchase visit and mandatory identification.

But good management also involves training the animal. Accustoming a camel to carrying a saddle and transporting young children, to entering a truck or a milking corridor, to behaving without aggression in a crowd and in front of the general public, and to participating in various sporting or recreational events requires preparing the animal for these different situations [106]. In camel countries, this learning is part of the long-standing, transgenerational cohabitation between camels and humans. From an early age, young shepherds learn to handle camels through direct experience and knowledge transmitted within families and local communities [105]. The absence of a tradition of camel breeding in European latitudes, and the sometimes lack of requirements on the part of new owners, may lead to unmanageable animals. Based on learning theory, including associative learning (classical and operant conditioning), non-associative learning (habituation and sensitization), and the appropriate use of reinforcement, different training methods have been developed to facilitate the acquisition of desired behaviors while improving handler safety and animal manageability and reducing fear and stress during human–camel interactions [102,106].

Ensuring camel welfare, therefore, has implications not only for good housing, good transport conditions, adequate feeding, and health protection, but also for building positive interactions between well-trained herders who understand their animals and well-trained camels that meet expectations. After good training on both sides, it is possible to obtain a nice, good-life companion from a camel, easier to manage.

Nevertheless, common welfare concerns in camels include not only inappropriate housing and unsuitable feeding, but also poor handling, inadequate transport conditions, and questionable slaughter practices. Coercive and rough handling has been reported in several camel countries, causing pain, injuries, and skin lesions [20,62,106]. Traditional tribal identification of camels through branding also remains widespread and represents an additional source of pain and injury [21,107]. This practice is less common in Western countries, where alternative identification methods, such as ear tags, boluses, or electronic devices, are more frequently used [108]. Inadequate transport conditions, including inappropriate loading and unloading procedures, vehicle conditions, journey duration, and loading density, may cause stress and compromise camel welfare [109,110]. Camels should therefore be transported in accordance with applicable animal transport legislation and international welfare standards, including those established by the World Organisation for Animal Health (WOAH), whose recommendations for the transport of animals by land explicitly include camels. In the European Union, camels also fall within the general scope of Council Regulation (EC) No 1/2005 on the protection of animals during transport, although the Regulation does not provide a comprehensive set of camel-specific transport requirements. Finally, slaughter of young or culled camels is currently uncommon in Europe and the USA. Should this practice become more widespread in the future, camel welfare during slaughter will need to receive the same attention as in other farm animal species; in the European Union, camels are covered by Council Regulation (EC) No 1099/2009 on the protection of animals at the time of killing, although this legislation does not provide camel-specific requirements.

## 6. Conclusions

The welfare of large camelids in Western countries appears to depend on two closely interconnected challenges: their capacity to acclimatize to environmental and husbandry conditions that differ substantially from those of their traditional rearing systems, and the ability of owners, caretakers, veterinarians and regulatory authorities to recognize and meet their species-specific needs. Western environments may offer several potential advantages, including greater availability of feed, water, shelter and veterinary care, but these conditions do not necessarily translate into better welfare. Nutrient-rich diets, reduced opportunities for locomotion and foraging, confinement, social restrictions, climatic conditions characterized by higher humidity, and unfamiliar management practices may introduce new welfare challenges.

Further research is therefore needed not only to understand camel acclimatization, but also to develop and optimize husbandry systems adapted to Western conditions while preserving camel-specific physiological, behavioral, and social needs. Furthermore, improved training of owners, handlers, and veterinary professionals, together with the development of appropriate regulations and guidelines that account for camel-specific requirements for keeping, transport, and slaughter, will be essential to support any future expansion of camel farming within a welfare-oriented framework.

## Data Availability

No new data were created or analyzed in this study. Data sharing is not applicable to this article.

## Abbreviations

The following abbreviations are used in this manuscript:

ECROA	European Camel Ranch Owners Association
NACROA	North American Camel Ranch Owners Association
GTV	Veterinary Technical Group
WOAH	World Organisation for Animal Health
